# Model predictive control optimisation using the metaheuristic optimisation for blood pressure control

**DOI:** 10.1049/syb2.12012

**Published:** 2021-02-14

**Authors:** Mohammad Reza Ahmadpour, Hamid Ghadiri, Saeed Reza Hajian

**Affiliations:** ^1^ Faculty of Electrical, Biomedical and Mechatronics Engineering Qazvin Branch, Islamic Azad University Qazvin Iran

## Abstract

Given the importance of high blood pressure, it is important to control and maintain a constant blood pressure level in the normal state. The main aim of this article is to design a model predictive controller with a genetic algorithm (GA) for the regulation of arterial blood pressure. The present study is an applied cross‐sectional study. In order to do this research, studies related to designing mathematical models for blood pressure regulation and mechanical models for heart muscle and pressure sensors are investigated. Then, a model predictive controller with GA is designed for blood pressure control. All control and design operations are performed in the MATLAB software. According to the viscoelasticity of blood, transducer, and injection set, we can assume the mechanical model as Mass, Spring, and Damper. Initially, the patient's blood pressure is lower than normal, and after controlling, the patient's blood pressure returned to normal. By using a GA‐based model predictive control (MPC), mathematical validation, and mechanical model, the patient's blood pressure can be adjusted and maintained. The simulation result shows that the GA‐based MPC offers acceptable response and speed of operation and the proposed controller can achieve good tracking and disturbance rejection.

## 1 INTRODUCTION

Haemodynamically, blood pressure is the force that imparts blood to the walls of the veins in which it flows. Normally, blood pressure increases and decreases continuously throughout the day, but if left elevated for a long time, it can damage the heart increasing the risk of heart attack [1, 2].

Hypertension is a serious medical condition that raises the risk of heart attack, stroke, and sightlessness leading to premature death. Of the approximately 1.2 billion people with high blood pressure, only 1%–5% of the population keeps it under control. An unhealthy diet, inactivity, alcohol, and red meat are the main reasons for high blood pressure [3, 4]. The above‐mentioned reasons and the complications of hypertension determine the blood pressure of a person which is especially important for the surgeons (during and after surgery). Hypertension often occurs in the early hours after surgery. In fact, some pills are used to treat high blood pressure after heart surgery or heart valve replacement. In recent years, with the development of intelligent systems to solve many complex problems, patient monitoring and medical data analysis have been developed using predictive systems‐based computer‐aided systems and impeller optimisation methods and many other intelligent techniques [5]. In this regard, due to uncertainty in various medical issues, model predictive control (MPC) is a powerful tool for intelligent decision‐making systems in the medical field and has an important role in medicine [6, 7, 8, 9, 10, 11, 12, 13, 14]. The combination of predictive controllers and intelligent methods is a very efficient and powerful way to control systems with limited inputs and outputs.

In fact, the controller is aware of the input and output restrictions and will in no way produce an input signal that violates the restrictions. Extensive research has been done on the combination of predictive model control and intelligent optimisation methods, which will be discussed further below. In Ref. [15], an MPC with particle swarm optimisation (PSO) is provided which according to the patient status, adjusts the drug injection to maintain mean arterial pressure (MAP) at the regulated value. On the other hand, in Ref. [16], a fractional‐Order PID controller has been designed to reduce MAP in cardiac patients after surgery. The fractional‐order PID controller is used to divide the injection of sodium nitroprusside (SNP) in a controlled approach into the patient's cardiovascular system to reduce high blood pressure.

Generally, the goal of controllers is to control the amount of drug being injected into the body, which in turn controls blood pressure and reduces complications during surgery and post‐operative treatment. In Ref. [17], a proportional‐integral‐derivative (PID) controller is designed to regulate CO and MAP by simultaneous injection of two drugs, both of which are nitroprussides (sodium and dopamine). The Ziggler–Nichols method is used to adjust the PID controller gate. Then, the PID gene is optimised using a GA and then the data is obtained from the deployment, elevation, and integrated square error as a target function. All medical instruments that connect to the body have viscoelasticity; additionally the tissue has the same as fats apart from muscles and veins. Also, the blood pressure of the body according to these specifications can be modelled as Spring, Mass, and Damper. Using mechanical elements to simulate blood pressure acts as the innovation. In Ref. [18], a fractional‐order PI controller is designed for regulating of Mean arterial blood pressure (MABP). Merits of the new design can be named as to give better disturbance rejection for sensitive and insensitive patients. Therefore, the technique proposed a simple robust controller. In Ref. [19], a new method using Back‐Propagation Neural Networks (BPNN) was used to calculate blood pressure, which according to the results shows that blood pressure is measured continuously. According to the investigations, eight features were extracted according to the time and frequency amplitudes related to the real‐time pulse signals measured by a pressure sensor to apply to the BPNN. It should be noted that this sensor is used to calculate systolic and diastolic blood pressure.

The drug injection control system is an electromechanical device that allows intravenous drug injection into the human body and increases its effectiveness by monitoring the rate and timing of drug release. Controlling moderate MAP and cardiac output during clinical practice is highly needed. The type‐2‐fuzzy logic controller based on PID is considered for MAP control and it is observed that the designed controller is robust under conditions of uncertainty, external disturbances, and noise [20].

Hence, the purpose of this study is to control blood pressure based on a combination of MPC with the metaheuristic optimisation method. The proposed approach improves the sensitivity of the blood pressure control system. Indeed, the MPC is an effective strategy for controlling non‐linear systems, and delay systems can optimise responses of the system when the system is under the states and control constraints. Due to using the GA method is expected to improve the responses.

The rest of this article is divided as follows. The blood pressure model and model prediction controller based on a genetic algorithm (GA) will be described in Sections 2 and 3, respectively, and the model prediction controller is optimised using a GA. The mechanical model of the heart muscle will be considered in Section 4. Section 5 is dedicated to present the simulation results and the resultant discussion. Finally, this article is concluded in Section 6.

## 2 BLOOD PRESSURE MODEL

Blood pressure is actually the average blood pressure over a heart period and is determined by the cardiac output, vascular resistance, and central venous blood pressure. Sustained control of MAP is important in the prevention of acute life‐threatening conditions such as stroke and the reduction of hypertensive diseases. Previous studies have shown that MAP is more accurate than the predicted metabolic syndrome among elderly people with hypertension determined by systolic, diastolic, and pulse pressure [21]. Today, the most important cause of death in a heart attack is hypoxia‐ischaemic brain injury [17]. However, if the MAP is below the threshold of automatic adjustment, it may lead to additional ischaemia and brain injury, and if the MAP is higher than the threshold of automatic adjustment, it may cause excessive strain which may lead to increased brain oedema and worsened brain injury [22]. Therefore, keeping blood pressure at an optimum range using the relationship between oxygen saturation and blood pressure in the brain tissue area is critical for survival in such patients [22].

**FIGURE 1:**
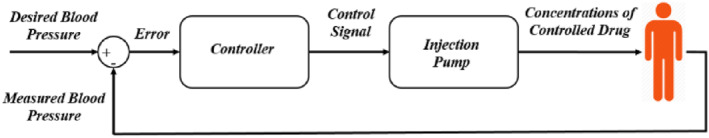
Structure of controlled drug delivery to adjust MAP

Hypotensive anaesthesia (anaesthesia by lowering blood pressure) is widely used in general surgeries, which results in decreased intraoperative bleeding and requires postoperative blood transfusion. On the other hand, this anaesthesia requires multiple injections of drugs to regulate key physiological variables such as alertness, heart rate, MAP, and respiratory rate [23]. The purpose of this control system is to reduce the MAP of the patient by adjusting the dose of nitroprusside and the drug. The controlled drug delivery structure to adjust MAP is shown in Figure 1.

In Figure 1, an injection pump, injection accessories, and body tissue as a mechanical model are explained. According to Figure 1, the controller based on the error between the set‐point and the patient's measured blood pressure provides a suitable control signal for proper injection of the drug into the injection pump.

In this form, the electrical signal is sent to a mechanical environment and is excited by the mechanical elements and eventually converted into an electrical signal. We considered a mechanical source as a source that is excited using an electrical signal. The mechanical source then generates a mechanical signal in a mechanical environment, and this signal is eventually converted to an electrical form and then used to control.

The MAP model is considered as follows [24]:

(1) $$\frac{\Delta MAP\left(s\right)}{SNP\left(s\right)}=\frac{K{\left(1+T_{3}s\right)}^{-\theta s}}{\left(\left(1+T_{3}s\right)\left(1+T_{2}s\right)-\alpha \right)\left(1+T_{1}s\right)}$$

where, ΔMAP is blood pressure changes, *SNP* is drug injection rate, *K* patient sensitivity to drug, *T*_1_, *T*_2_, and *T*_3_ drug effect time constants, *θ* system delay, and *α* The constant is the circulation of the drug.

## 3 MODEL PREDICTIVE CONTROL

MPC is a control technique, that is used to control a process which includes a set of constraints. MPC is an advanced controller for controlling industrial processes in which the physical constraints on the system can be considered during design. MPC operates based on iterative optimisation of a cost function with a finite horizon at each sampling time, and the control action generates based on the past, present, and future information.

**FIGURE 2:**
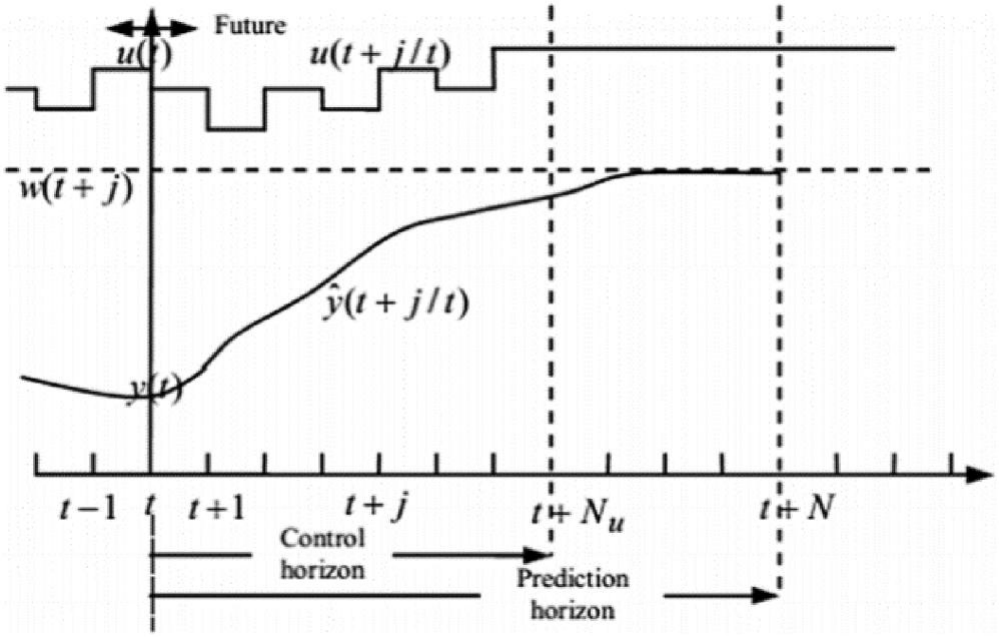
The strategy of model predictive control [25]

Figure 2 is shows the MPC strategy. It should be noted that the first step of the control strategy is implemented, and the plant state is sampled again, and the calculations are repeated starting from the new current state, and a new control and the path of the new predicted state are obtained.

The prediction horizon is continuously moved forward, and for this reason, MPC is also called the receding horizon control.

**FIGURE 3:**
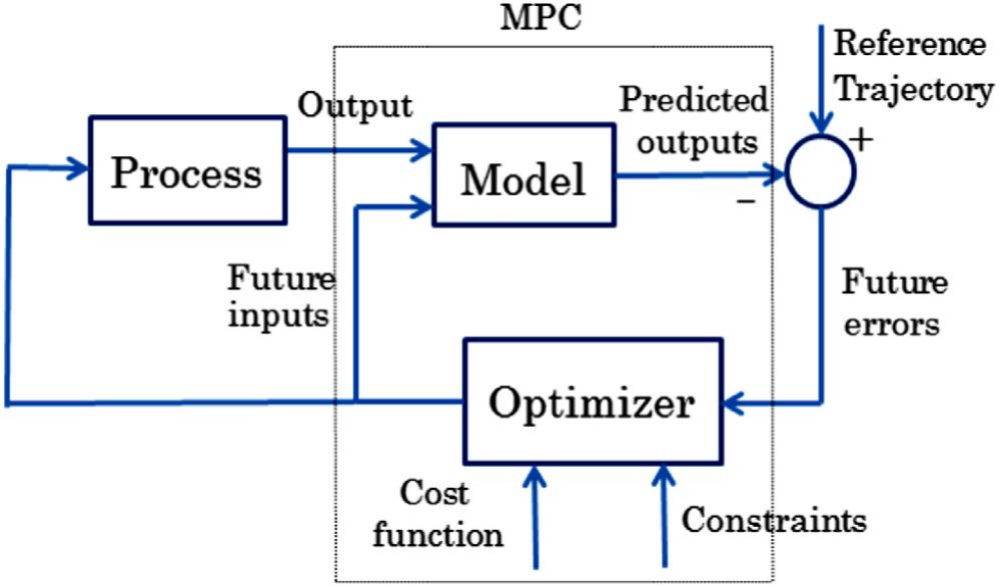
Block diagram of MPC [25]

MPC has been used as a very practical and successful control method with a simple configuration and it also has been known as a practical controller, that is it able to consider multivariate systems and control the online optimization process. The main structure of the MPC strategy with horizon control is shown in Figure 3 using the following method [25]:

1. At every instant, the behaviour indicated by the plant as well as future plant outputs are calculated in the first place by using a dynamic process model based on the most recent observation from system inputs and outputs.
2. Control signal inputs are calculated by minimising the signal error tracking the difference between the predicted output and the signal of the desired path to follow the trend as much as possible by considering the objective function and constraints.
3. While other control signals are removed as a result of the following sampling instant, merely the first control signal is applied within the plant.
4. The updated value went along with Step 1.

The cost function and would be demonstrated as follow:

(2) $$J=\sum_{i=1}^{P}\mu_{i}{\left(\hat{y}\left(t+i|t\right)-w\left(t+i\right)\right)}^{2}+\sum_{i=1}^{M}\lambda_{i}{\left(u\left(t+i\right)-u\left(t+i-1\right)\right)}^{2}$$

where, *P* is the prediction horizon, *M* is the control horizon, *μ*_*i*_ and *λ*_*i*_ are weighting coefficients. $\hat{y}\left(t+i|t\right)$, *w*(*t* + *i*), *u*(*t* + *i*) are predicted output, desired output, and future control signal, respectively, and the constraints considered as below:

(3) x(t+1)=f(x(t),u(t)),

(4) y(t)=g(x(t),u(t)),

(5) $$\underline{u}\leq \;\|u\left(t)\right\|\leq \bar{u},$$

(6) $$\underline{x}\leq \;\|x\left(t)\right\|\leq \bar{x}.$$

where, $\underline{u}$, $\bar{u}$ are lower bound and upper bound of signal control, $\underline{x}$, $\bar{x}$ are lower bound and upper bound of state of the system, respectively.

The main goal is to minimise the cost function Equation (2) by considering constraints Equations (3)–(6).

## 4 GENETIC ALGORITHM

On the basis of natural selection theory, GA is a means of resolving constrained and unconditioned (unrestricted) optimisation problems (issues), which constantly alter the population of single answers. People from the most recent generation at GA such as parents, are chosen which are later utilised to make children. It is important to note that these children themselves are members of the upcoming generation. Among generations that appear consecutively, the number of responses is seen to have developed to an optimal (optimum) response. Considering other features GA, it can be said that several optimisation problems for which standard optimisation algorithms were not compatible to solve, could be resolved [26].

At each stage, GA uses three basic rules to create the next generation of the current population:

- Selection rules, select individual answers called parents.
- Displacement laws, combine parental characteristics to form their next‐generation child.
- Mutation laws, randomly apply changes to one (or both) parents to form the offspring of the next generation.

The differences between the GA and the classical derivation‐based optimisation algorithm are summarised in Table 1.

**TABLE 1:** Comparison of the classical algorithm with GA

Classical algorithm	Genetic algorithm
In each computational step, a point is created. The sequence of these points tends to the optimal answer.	In each computational step, a set of points is created. The best point in the population tends to the optimal answer.
The next point determines the sequence with definite calculations.	The next‐generation population is determined by calculations using random numbers.

One of the key benefits of using GA‐based MPC is its ability to manage a variety of objective functions and processing models without changing the structure of the controller.

## 5 MODEL PREDICTIVE CONTROL STRUCTURE BASED ON GA

**FIGURE 4:**
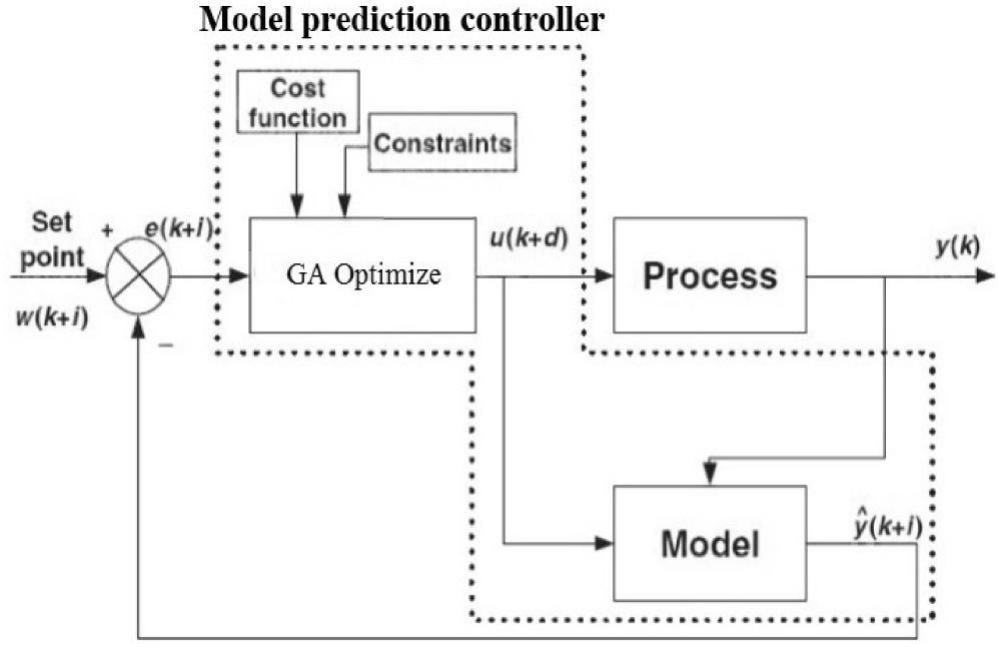
Model predictive control structure based on GA [27]

GA‐based control uses the process model to search for control movements, which meet process constraints and optimise a cost function. On the other hand, due to the high computational load in MPC and its slowness, the GA‐MPC method can increase the speed of the system and significantly reduce the computational volume. MPC structure based on GA is shown in Figure 4 [27].

Adjusting the input weight matrix can greatly fluctuate the closed‐loop control system. Therefore, using the MPC‐GA approach can reduce these fluctuations to some extent and improve the system response. The following steps describe the operation of the GA‐MPC algorithm:

1. While making use of the process model, examine process outputs.
2. By doing a GA search in order to find optimal control motion through which the cost function is increased to an optimum level and the limitations (constraints) of the process are satisfied.
3. The optimal control movements created in Step 2 are implemented to the process.
4. Step 1 to three applies to time.

**FIGURE 5:**
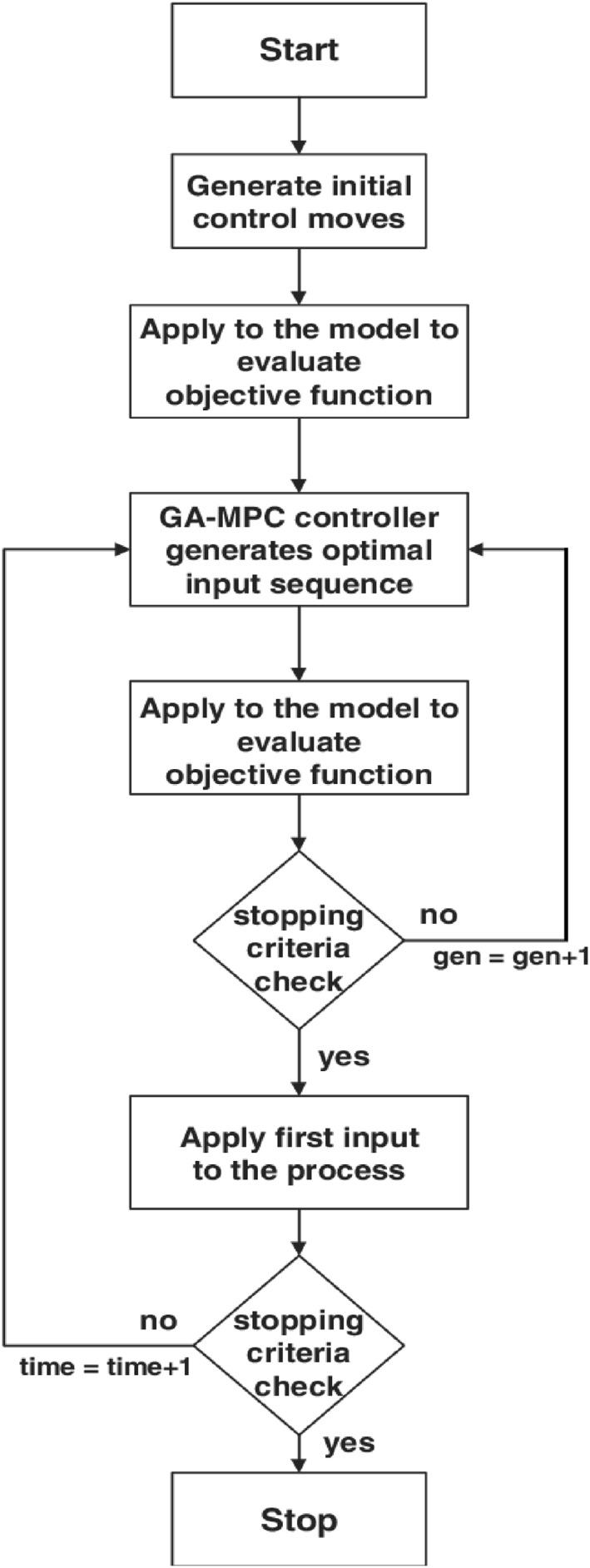
Flowchart GA‐based MPC approach

The flowchart of the algorithm of the GA based on MPC is shown in Figure 5.

## 6 THE MECHANICAL MODEL OF THE HEART MUSCLE

The cardiovascular system is actually a mechanism of blood transfusion that delivers nutrients to the tissues and organs of the body and eliminates toxic waste.

This cycle involves the heart, and most importantly the heart muscle that circulates blood and blood vessels, which transfers blood to various organs as well as the lung, thereby activating the pulmonary arteries to exchange oxygen and carbon dioxide in the lungs.

Blood is transmitted from the aorta to the arteries, arteries, and capillaries. The blood flow to the cardiovascular system is under the laws of mass, motion, and interaction with the arterial wall. Choosing the right model size depends on the goals and accuracy you are looking for. Parametric models of distribution operate mainly on uniform distribution and take into account fundamental variables such as pressure, flow, and volume of the muscle to each muscle or organ at any given moment.

This approach provides conventional differential equation heart muscle modelling to evaluate the pervasive distribution of pressure, flow, and blood volume across a range of physiological conditions. Mathematical models of blood flow, with their non‐destructive properties, facilitate the study of pathological and physiological forms, and the calculation of pressure and flow profiles can be part of potential future diagnostic tools.

On a patient‐specific basis, profiles can be compared with physiological cases showing normal or pathological blood flow. Investigating the dynamics of neuromuscular reflex movements can provide valuable insights into the status of patients with hypertensive disorders. Consider a patient who sits comfortably with his or her shoulder and elbows with adjustable support and does not perform any activity that increases or decreases heart rate and is in a constant position. Then, over time t=0, the pressure suddenly increases and the heart muscle begins to act quickly. *M*_*x*_ Indicates a momentary change in heart muscle function, which is considered here to be an increase in pressure. *M* represents an effort made in response to increased pressure by the heart muscle. Given these conditions, we will rewrite Newton's Second Law as follows [15]:

(7) $$M_{x}\left(t\right)-M\left(t\right)=J\ddot{\theta }$$

where, *J* is the point where the heart resides.

**FIGURE 6:**
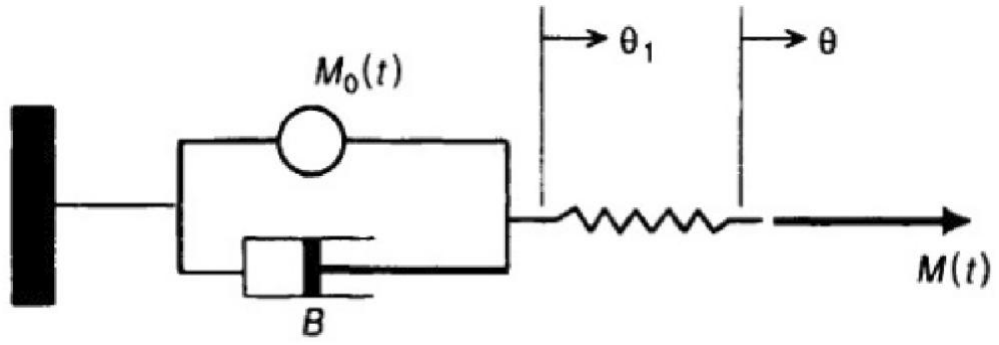
Heart muscle model

The heart muscle response consists of systole and diastole, and in order to avoid complexity, we assume that the muscle contraction produced by *M*_*x*_ is simplified to a mechanical model of the heart muscle as follows (Figure 6) [28]:

It should be noted that in this mechanical system, muscle contraction, transducer and its set can be summed as mas, spring, and damper, which can show the vascularity of the cardiac muscle, transducer and its set. *M* is regarded as a force while it is in torque. Subsequently, the ‘displacement’ action is actually the reaction of the heart muscle with angular variations *θ* to *θ*_1_. Likewise, the degree of myocardial stiffness (*k*) and the viscosity change (*B*) parameter are considered as constant units. The equations of motion for the muscle model are:

(8) $$M\left(t\right)=k\left(\theta -\theta_{1}\right)$$

and considering (*B*)

(9) $$M\left(t\right)=M_{0}\left(t\right)+B{\dot{\theta }}_{1}$$

*M*_0_ is a torque that is produced by the heart muscle at the same conditions and *M*_0_(*t*) is actually a temporal operator. By combining the two equations above, a kinetic equation is obtained that determines the dynamics of the system as well as *θ* changes in the torque applied by hypertension. The equation will be as follows:

(10) $$\frac{BJ}{k}\dddot{\theta }+J\ddot{\theta }+B\dot{\theta }=M_{x}\left(t\right)-M_{0}\left(t\right)$$

As stated in the mechanical model of the heart, muscle tissue is modelled by mechanical elements. In this model, the spring is used as muscle tissue tension, the damper as the spring‐resistance, and the spring stabiliser.

We draw the concern to this point that damper can mention the resistance of the injection, muscle and injection liquid, spring and mass are noted as viscoelacity of these three items. Assume mass, spring, and damper in one block box. In this block box, mass and spring can be named for the added viscoelacity and damper also be added to the resistance system. Block box (Mass, Spring, and Damper) is drawn, fixed, and simulated in Simulink toolbox of MATLAB. Simmechanic is one part of SIMULINK which can place the sensor and monitor the measurement online. Sensors in SimMechanic can produce mechanical signals. We transfer the mechanical signals to electrical signals to m‐file and run the mechanical model in Simmechanic, and transfer mechanical data to m‐file that can be noted as innovation; additionally we also run one mechanical black box as the system's resistance and viscoelacity has merits and demerits that will be explained.

## 7 SIMULATION RESULTS AND COMPARISONS

Regular and multi‐parameter implementations since the control system performance simulation time aremainly related to the execution of the control algorithm, and it is sufficient to compare the execution time of the control algorithms to compare the total run time of the simulation for different controllers.

### 7.1 Trajectory tracking

For the first simulation, it is assumed that the model of the system operates as follows [15]:

(11) $$\begin{array}{l} G_{P}\left(s\right)=\frac{\Delta MAP\left(s\right)}{SNP\left(s\right)}\;\\ =\frac{5\left(1+30s\right)e^{-60s}}{1+130s+4600s^{2}+30000s^{3}} \end{array}$$

**FIGURE 7:**
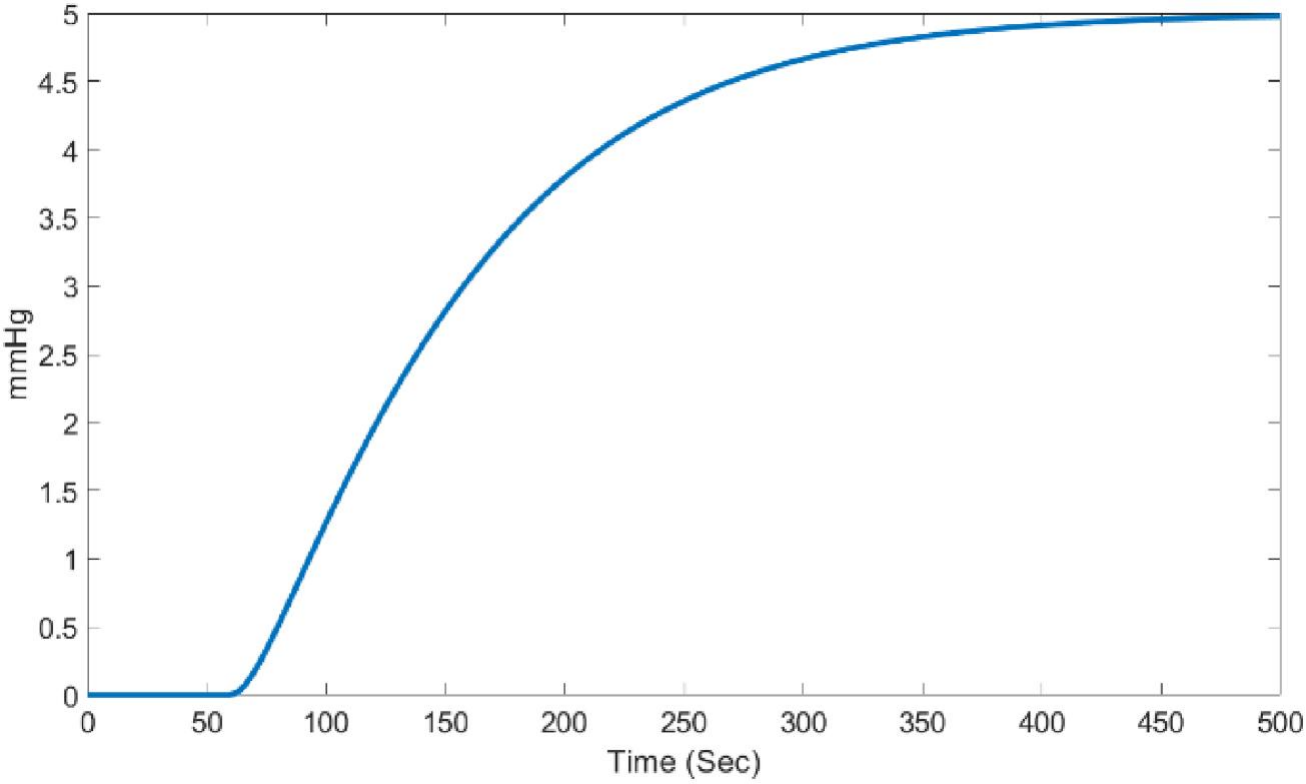
The step response of the system

Simulation is performed in MATLAB and Simulink environments It can be assumed that the runtime in other software environments is proportional to the time taken in MATLAB software. Figure 7, shows the unit step response of the open‐loop system.

The first design step is changing the continuous model to discrete model. First, we discretise the continuous system using zero‐order hold with a sampling time of 1 ($T_{s}=1$). Therefore, the discrete system will be as follows:

(12) $$G_{P}\left(z\right)=z^{-60}\frac{b_{2}z^{2}+b_{1}\;\text{z}+b_{0}}{z^{3}+a_{2}\;z^{2}\;+\;a_{1}\;\text{z}+a_{0}}$$

where,

$$b_{0}=-\;0.002233,\;b_{1}=-\;1.543\times 10^{-5},\;b_{2}=0.002403,$$

$$a_{0}=-\;0.8578,\;a_{1}=2.712\;,\;a_{2}=-\;2.854$$

**FIGURE 8:**
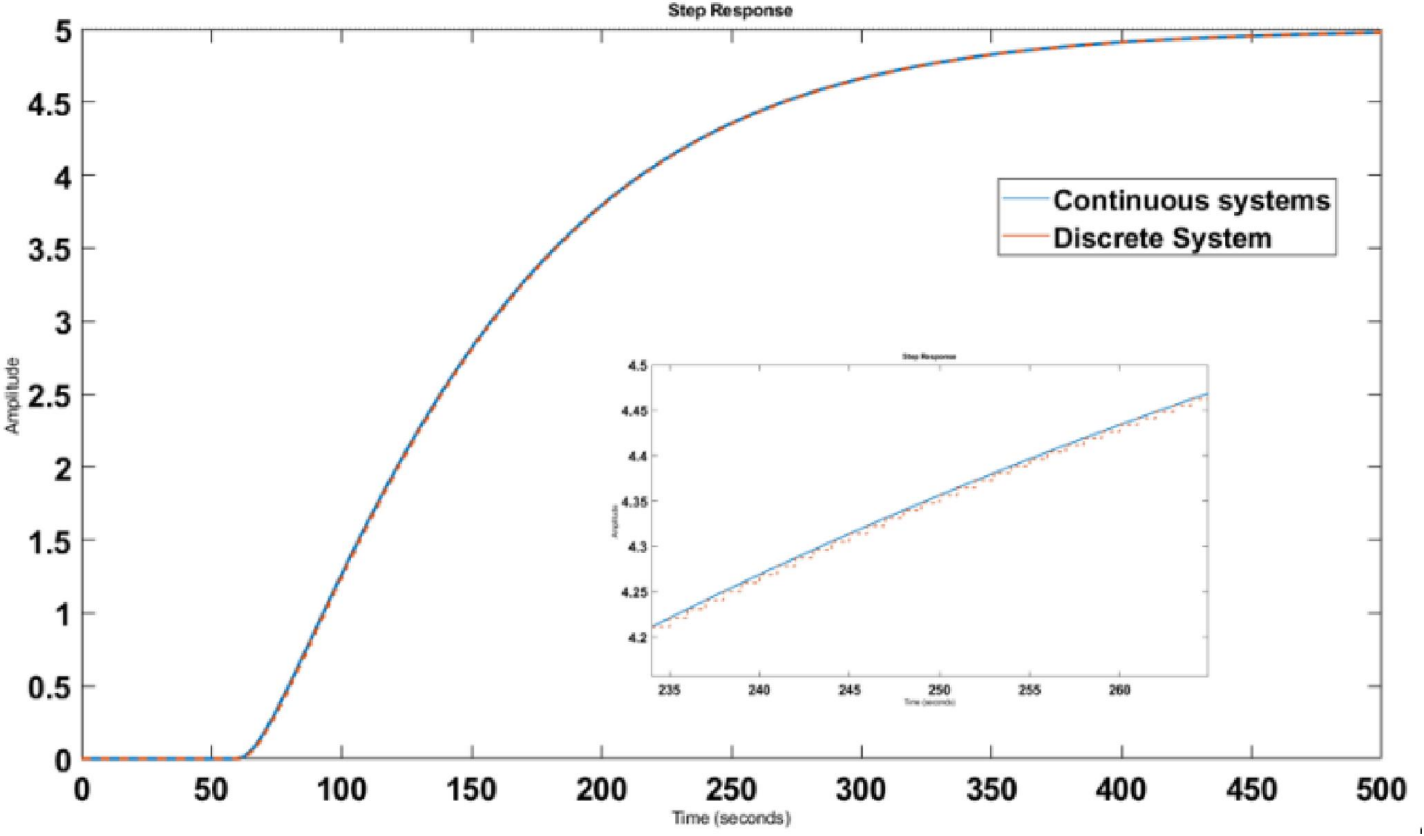
The step response of the discrete system

Figure 8 shows the unit step response of the open‐loop discrete system. According to Equation (6), the system output will be as follows:

(13) $$y\left(k\right)=-a_{2}\;y\left(k-1\right)-\;a_{1}\;y\left(k-2\right)-a_{0}y\left(k-3\right)+b_{2}u\left(k-61\right)+b_{1}\;u\left(k-62\right)+b_{0}u\left(k-63\right)$$

Now, the design of controller parameters will be discussed. In cost function Equation (2), we consider M=2, $\mu_{i}=1$, and $\lambda_{i}=0.1$. Given the values presented above, and considering the cost function relationship, this function would be:

(14) $$J=\sum_{i=1}^{P}{\left(\hat{y}\left(t+i|t\right)-w\left(t+i\right)\right)}^{2}+\sum_{i=1}^{2}0.1{\left(u\left(t+i\right)-u\left(t+i-1\right)\right)}^{2}$$

The limitation of the drug infusion rate is the constraint of the proposed controller [29]:

(15) $$u\leq 2\left(\frac{ml}{h}\right)$$

Given that the GA is involved in optimisation, the following figure shows the convergence performance of this algorithm in each iteration. Due to the long simulation time, these quantities gradually converge to zero. This demonstrates the high capability of the algorithm to find the optimal point without the need for sophisticated computation. The parameters of the GA adjustment are summarised in Table 2.

**TABLE 2:** Genetic algorithm adjustment parameters

Parameters	Values
Mutation probe	0.005
Crossover probe	0.7
No. of generations	10
Population size	100
Insertion rate	1

**FIGURE 9:**
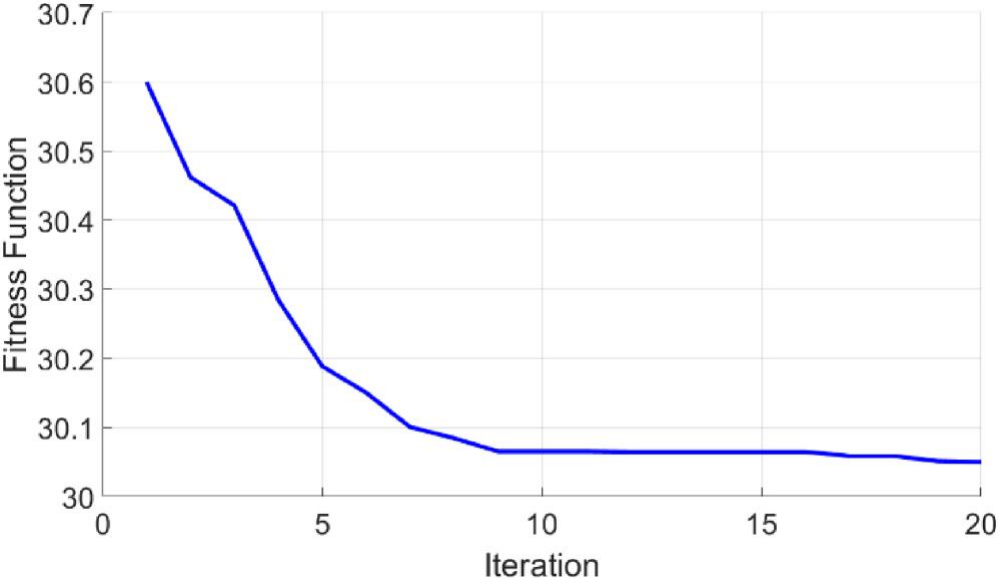
GA convergence process

In Figure 9, the convergence process of GA is shown. Only after several iterations, the fitness value reaches the smallest.

**FIGURE 10:**
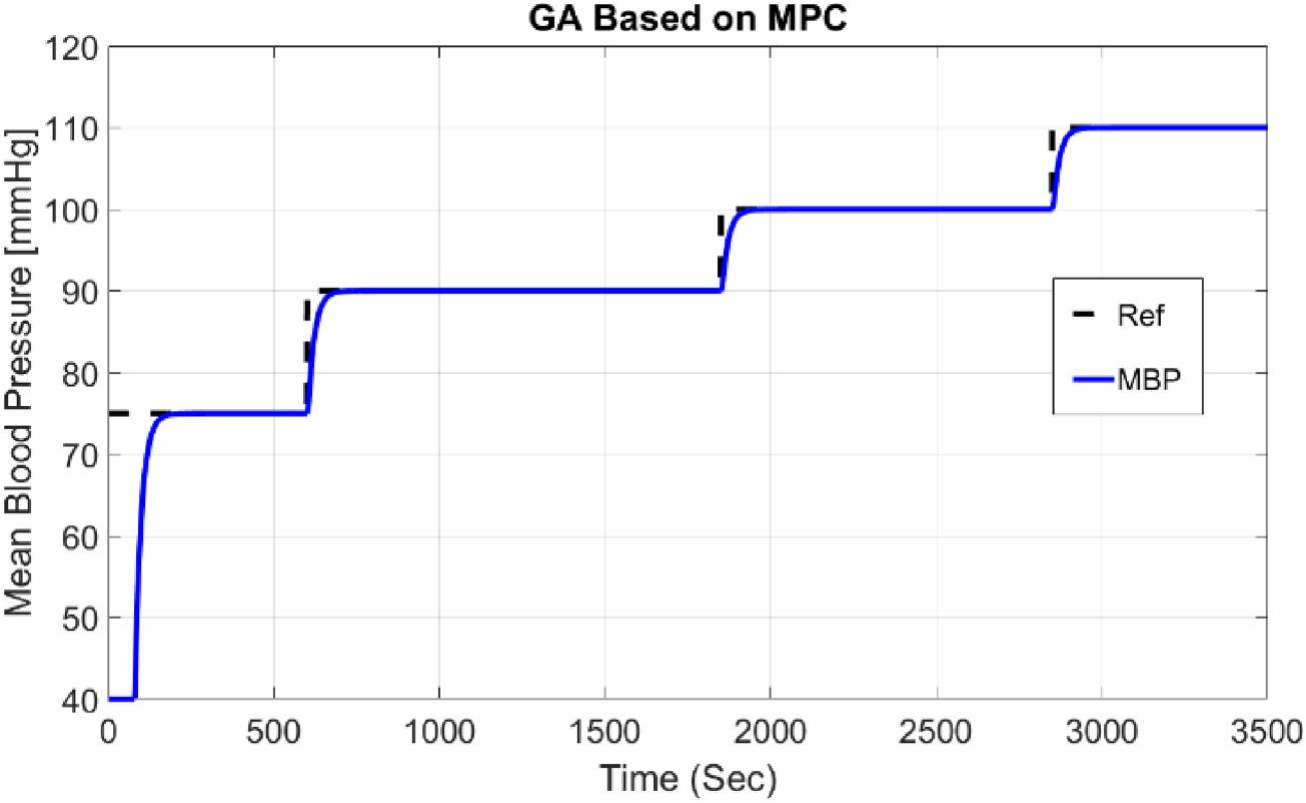
The output response of the system with GA‐based MPC (*P* = 20, *M* = 2)

We then plot the complete system response presented in Figure 10 for the desired output of the step signal. Figure 10 shows the response of the controlled system. As we can see in Figure 10, the system response follows the desired output.

The initial visit blood pressure for the patient is considered 40 mmHg. As shown in Figure 10, the system response follows the desired output and in the first second reaches normal blood pressure.

### 7.2 Investigation of the effect of parameter P

Investigating the effect of parameters is a reliable way to check how a control approach works, and by applying the changes to each one, we can find out how the control system works and use it in designing or improving previous designs.

**FIGURE 11:**
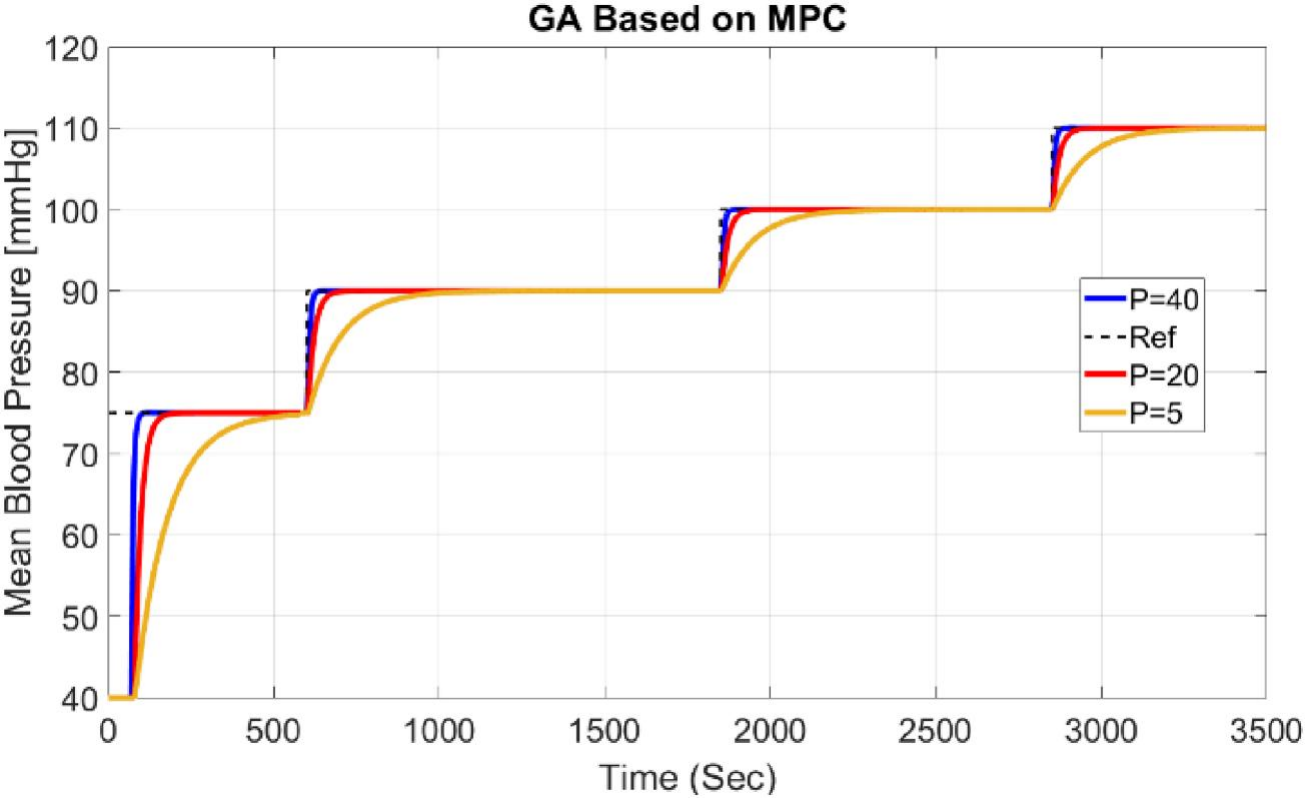
MAP for different values of Prediction Horizon (*P*)

Overall, this review provides a comprehensive overview of the performance of the controller under different conditions. In Figure 11, the influence of the prediction horizon (*P*) on the system response is shown. As the horizon increases, the computational volume increases, but the signal becomes softer and has a lower jump than lower values. On the other hand, this increase makes the system slower. As the prediction horizon decreases, the system speed increases, but on the contrary, the amount of jump increases.

Additionally, Integral Absolute Error (IAE), Integral Square Error (ISE), and Mean Square Error (MSE) error criteria are presented for different values of *P* in Table 3.

**TABLE 3:** Error criteria for different values of *P* and *M* = 2

Prediction horizon (*P*)	ISE	MSE	IAE
*P* = 5	3.02	0.107	5.25
*P* = 20	2.10	0.011	4.42
*P* = 40	2.14	0.012	4.65

### 7.3 Disturbance effect

**FIGURE 12:**
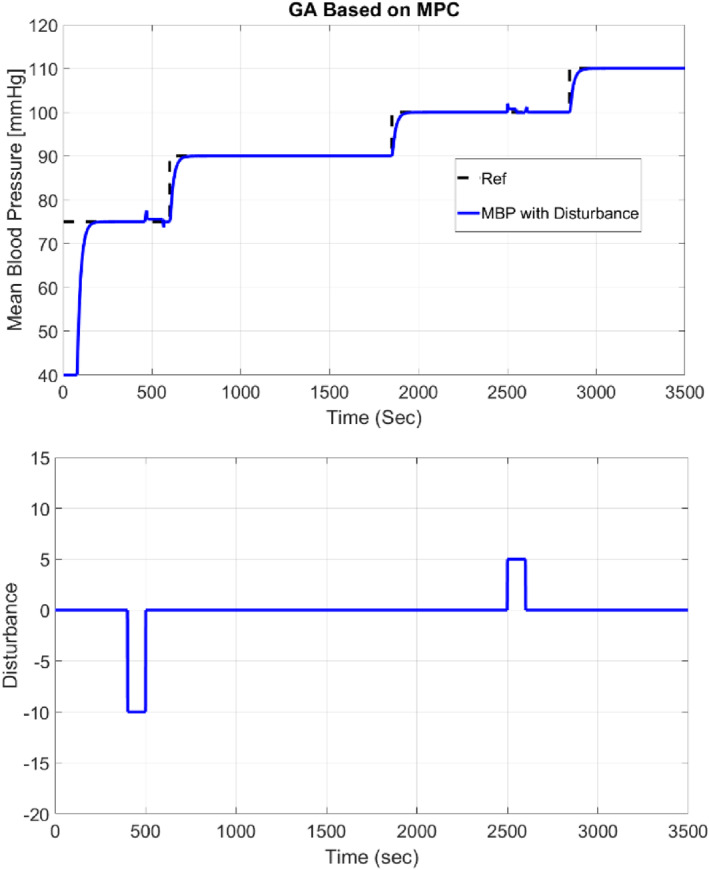
The effect of drug disturbance on the patient

The following is a review of the effect of the perturbation on the patient, which is the effect of diuretics that is mainly used in patients with renal impairment, and its major side effect is the reduction of the patient's blood pressure. The disturbance is considered to be sinusoidal and also can be noted as the heartbeat, injection device, percentage of injection material and nervous system. The amplitude of this perturbation is 10. Figure 12 demonstrates that the effect of drug‐induced perturbation has been eliminated.

Also, Table 4 shows IAE, ISE, and MSE error criteria with and without disturbance.

**TABLE 4:** Error criteria in the presence of disturbance (*M* = 2, *P* = 20)

Disturbance effect	ISE	MSE	IAE
Without disturbance	2.10	0.011	4.42
With disturbance	2.20	0.014	4.63

**FIGURE 13:**
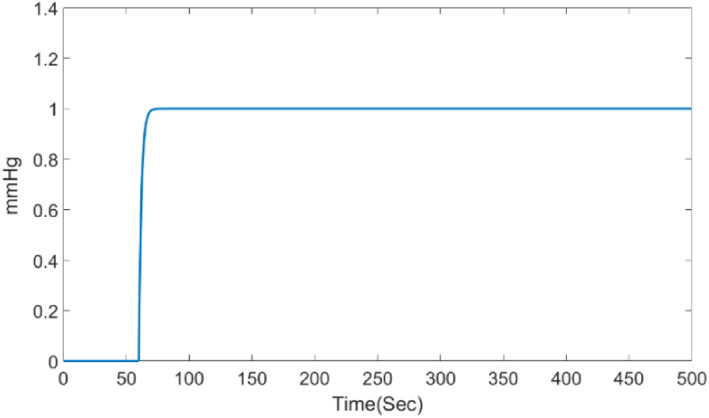
Output response (MAP) of the system with GA‐based MPC (*P* = 20, *M* = 2)

**FIGURE 14:**
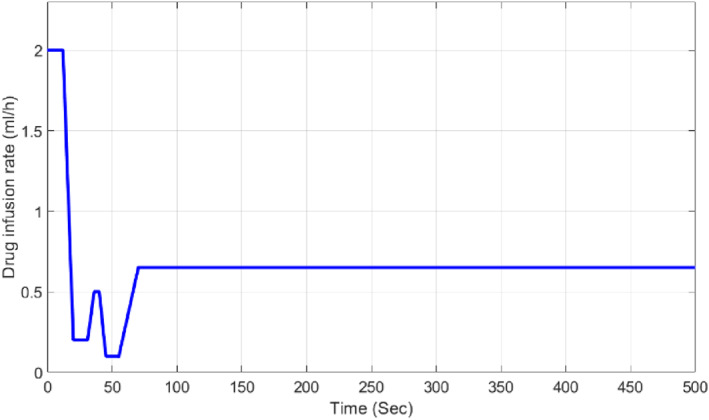
Control signal corresponding with GA‐based MPC to set point responses

In this section, we consider the unit step as the reference value. The output (MAP) and the control signal (Drug infusion rate) of the proposed GA‐based MPC control system are shown in Figures 13 and 14.

Figure 14 shows that the constraint of the control signal is satisfied according to Equation (15).

### 7.4 Comparison of GA‐based MPC with PID controller, MPC, and PSO‐based MPC

**FIGURE 15:**
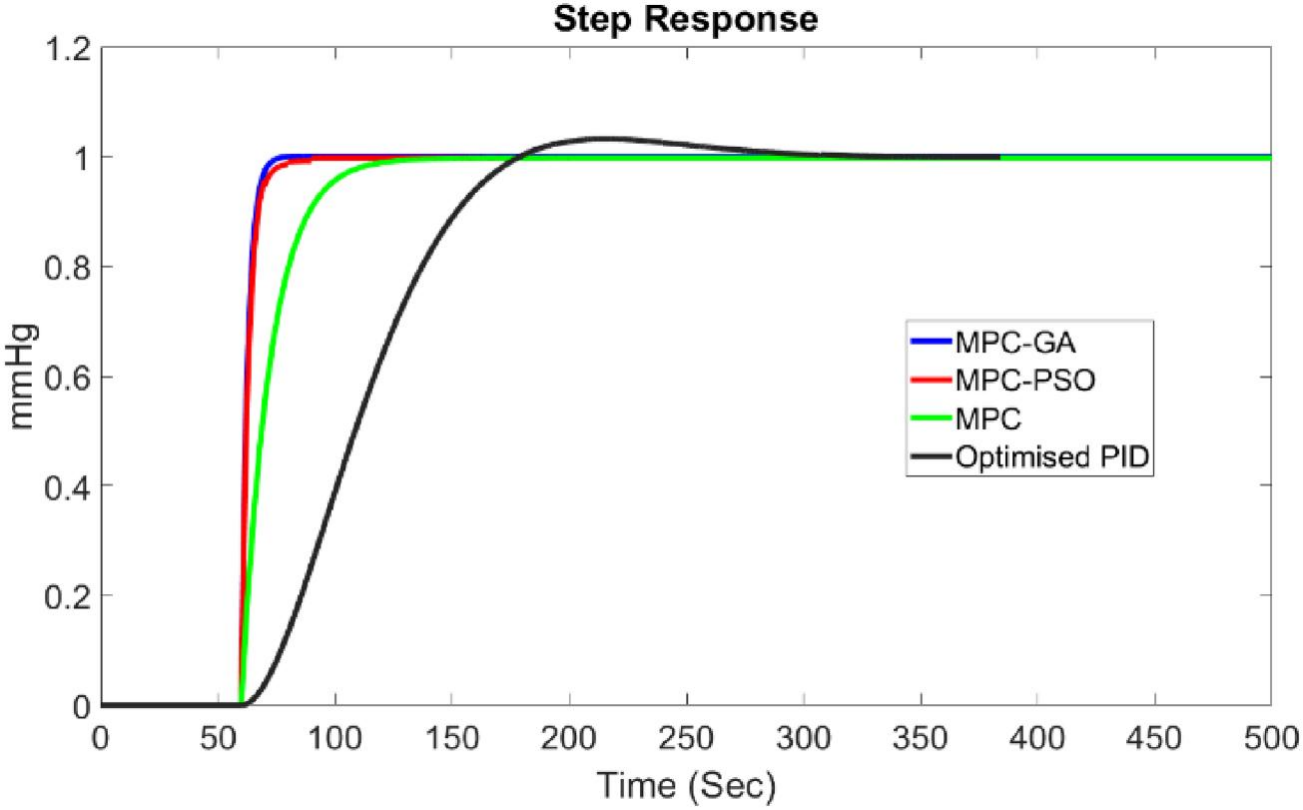
The step response (MAP) for MPC‐GA, MPC‐PSO MPC, and the PID controller

The comparison in Figure 15 proves that the performance of the GA‐based MPC is much better than the optimised PID controller [24], PSO‐based MPC, and MPC.

It should also be noted that the system always responds faster than other controllers. This is one of the main benefits of MPC. With significant advances in system response, this control approach is applicable to a wide range of patients.

Additionally, the comparison between the performances of the controllers is shown in Table 5.

**TABLE 5:** Comparison between the performances of the controllers

Control method	IAE	ISE	MSE	Execution time (s)
GA‐based MPC	4.42	2.10	0.011	66.9531
PSO‐based MPC	4.54	2.19	0.018	49.032
MPC	6.21	3.57	0.72	173.672
PID	7.12	4.25	0.98	20.2

## 8 CONCLUSION

Among the advanced control methods, MPC has been in place for decades, as the most successful advanced process control method has found a good place in various industries.

MPC can be described, firstly, by foretelling the future output of the system, and secondly, by calculating future output signal online which is carried out by means of bringing the target function to the lowest level under state and input constraints. On the final stage, they apply to real systems and then the stages covered so far are again duplicated by measuring state, output, and input variables.

Therefore, the presence of a proper model of the real system is a prerequisite for the design of interpolation control. The approaches that are nowadays more favoured are the combination of this controller with meta‐heuristic methods and optimisation algorithms. Therefore, in this study, hypertension control strategy using a predictive control technique and its combination with GA are presented. The main focus of this article is to evaluate the performance of the GA based on MPC strategy that has been used to achieve optimal SNP drug infusion rate to regulate MAP and also to apply this method to the cardiac muscle mechanical model and using this mechanical model is the main difference compared with Ref. [15]. The GA optimisation method was used to adjust the controller parameters. After lowering blood pressure to a normal control value, the MAP returns to normal and remains stable at approximately 80 mm Hg by performing drug injection and taking into account the physical limitations of the injection pump. The results indicate that the GA‐based MPC has better performance than PID controller with the delay and the limit of the control signal.

As can be seen from the results of parameter changes, the large forecast horizon will provide more stability for the closed‐loop system. Therefore, the idea of the forecast horizon has a significant impact on the design of a stable predictor controller. Of course, choosing large quantities for the forecast horizon increases the optimisation calculations. Changes to the control horizon also allow the control of the large horizon control to be activated, making the system slower and less mobile for smaller values of the control horizon. In general, this study focuses on the control of MAP. Other haemodynamic variables were not considered. In order to bring the results of this research to real and practical blood pressure control systems, the heart muscle mechanical model has been used and mainly mechanical systems have many limitations in their performance, as well as its configuration and relationship with the controller which actually has an electrical nature put a lot of restrictions. The simulation results achieve the main objectives and the algorithms provide good performance.

However, the following suggestions for future research are available:

- Applying and testing the control approach presented in clinical trials using animals.
- Implement interactive MIMO control instead of using SISO control loop.
- Provide model patient models to further manage physiological parameters such as mean pulmonary artery pressure as output and adjust it by injecting phenylephrine as input and also considering patient stress and age as impactful parameters on blood pressure.

Obtain real patient information and use a neural network to obtain a non‐linear model that best describes the patient's response to various drugs.
